# Examining geographical disparities in the incubation period of the COVID-19 infected cases in Shenzhen and Hefei, China

**DOI:** 10.1186/s12199-021-00935-3

**Published:** 2021-01-18

**Authors:** Zuopeng Xiao, Wenbo Guo, Zhiqiang Luo, Jianxiang Liao, Feiqiu Wen, Yaoyu Lin

**Affiliations:** 1 Harbin Institute of Technology, Shenzhen, Guangdong China; 2 Shenzhen Children’s Hospital Affiliated with China Medical University, Shenzhen, Guangdong China; 3 School of Geography and the Environment, University of Oxford, Oxford, UK

**Keywords:** Geographical disparities, Incubation period, Travel history, Meteorological conditions, COVID-19

## Abstract

**Background:**

Current studies on the COVID-19 depicted a general incubation period distribution and did not examine whether the incubation period distribution varies across patients living in different geographical locations with varying environmental attributes. Profiling the incubation distributions geographically help to determine the appropriate quarantine duration for different regions.

**Methods:**

This retrospective study mainly applied big data analytics and methodology, using the publicly accessible clinical report for patients (*n* = 543) confirmed as infected in Shenzhen and Hefei, China. Based on 217 patients on whom the incubation period could be identified by the epidemiological method. Statistical and econometric methods were employed to investigate how the incubation distributions varied between infected cases reported in Shenzhen and Hefei.

**Results:**

The median incubation period of the COVID-19 for all the 217 infected patients was 8 days (95% CI 7 to 9), while median values were 9 days in Shenzhen and 4 days in Hefei. The incubation period probably has an inverse U-shaped association with the meteorological temperature. The warmer condition in the winter of Shenzhen, average environmental temperature between 10 °C to 15 °C, may decrease viral virulence and result in more extended incubation periods.

**Conclusion:**

Case studies of the COVID-19 outbreak in Shenzhen and Hefei indicated that the incubation period of COVID-19 had exhibited evident geographical disparities, although the pathological causality between meteorological conditions and incubation period deserves further investigation. Methodologies based on big data released by local public health authorities are applicable for identifying incubation period and relevant epidemiological research.

## Introduction

The outbreak of the 2019 novel coronavirus (COVID-19) has rapidly spread across China and then to several other countries/regions in the month since it emerged in Wuhan in December 2019. Although the vast majority of the initial infected cases were reported in China, the sudden public health crisis caused by this novel respiratory virus poses a serious and imminent threat to the global community [1]. According to the World Health Organization, this virus primarily spreads by respiratory droplets, saliva, and nasal discharge. After being infected, the mild symptoms include a runny nose, sore throat, cough, and fever, which in severe cases can lead to pneumonia or dyspnea [2].

Compared to the clinical symptoms, which gradually are well established, debates on the incubation period are more diversified. The incubation period refers to the time gap between infection and the onset of clinical symptoms. As a key epidemiological parameter, the incubation period has been extensively investigated by clinical studies on COVID-19 [3–10]. These studies, however, all depicted a general incubation period distribution based on the available cases and did not examine whether the incubation period distribution varies across cases living in different geographical locations with varying environmental attributes. A few studies have confirmed the effects of air conditions on the viability of the SARS [11–14]. For instance, Chan et al. described the prevalence of the SARS coronavirus in the spring in subtropical Asia areas with an average temperature of 22-25 °C and relative humidity of 40-50% [12]. Based on the strong similarity between the SARS virus and COVID-19 [15], it is reasonable to hypothesize that there exist geographical effects of meteorological conditions on COVID-19 and further examine whether infected persons in different places experience varying incubation periods.

Hence, the research question in this study is how geographical factors affect the incubation period of COVID-19 and whether that period varies by location. Incorporating geographical variance into an analysis of the incubation period could refine the appropriate duration of quarantine in different locations and determine how many days should be included in the analysis of contacts made by infected cases. We also explored the applicability of potential big data released by local public health authorities in understanding the epidemiological process.

## Data and methods

### Data source and research area

This retrospective study mainly used publicly accessible data released by local public health authorities. As one measure to address COVID-19, local public health authorities in China have been required to release information on the infection situation online every day since the middle of January. Most city/county public health authorities provide the day-to-day aggregated numbers of infected cases. However, the public health authorities of some cities release brief clinical reports (including travel routes and clinical features) for each confirmed infected case. Among them, two cities, Shenzhen in the subtropical zone and Hefei in the temperate zone were selected because they released the to-some-extent detailed data at their earliest stage. The two cities have significantly different geographical and climate characteristics (Table 1).

**Table 1:** Basic information on Shenzhen and Hefei

City	Shenzhen	Hefei
Permanent population	10.77 million	7.96 million
Geographical zone	Subtropical	Temperate
Latitude	22° 32′ N	31° 86′ N
Average temperature in January		
Min	20 °C	8 °C
Max	13 °C	0 °C

**Fig. 1:**
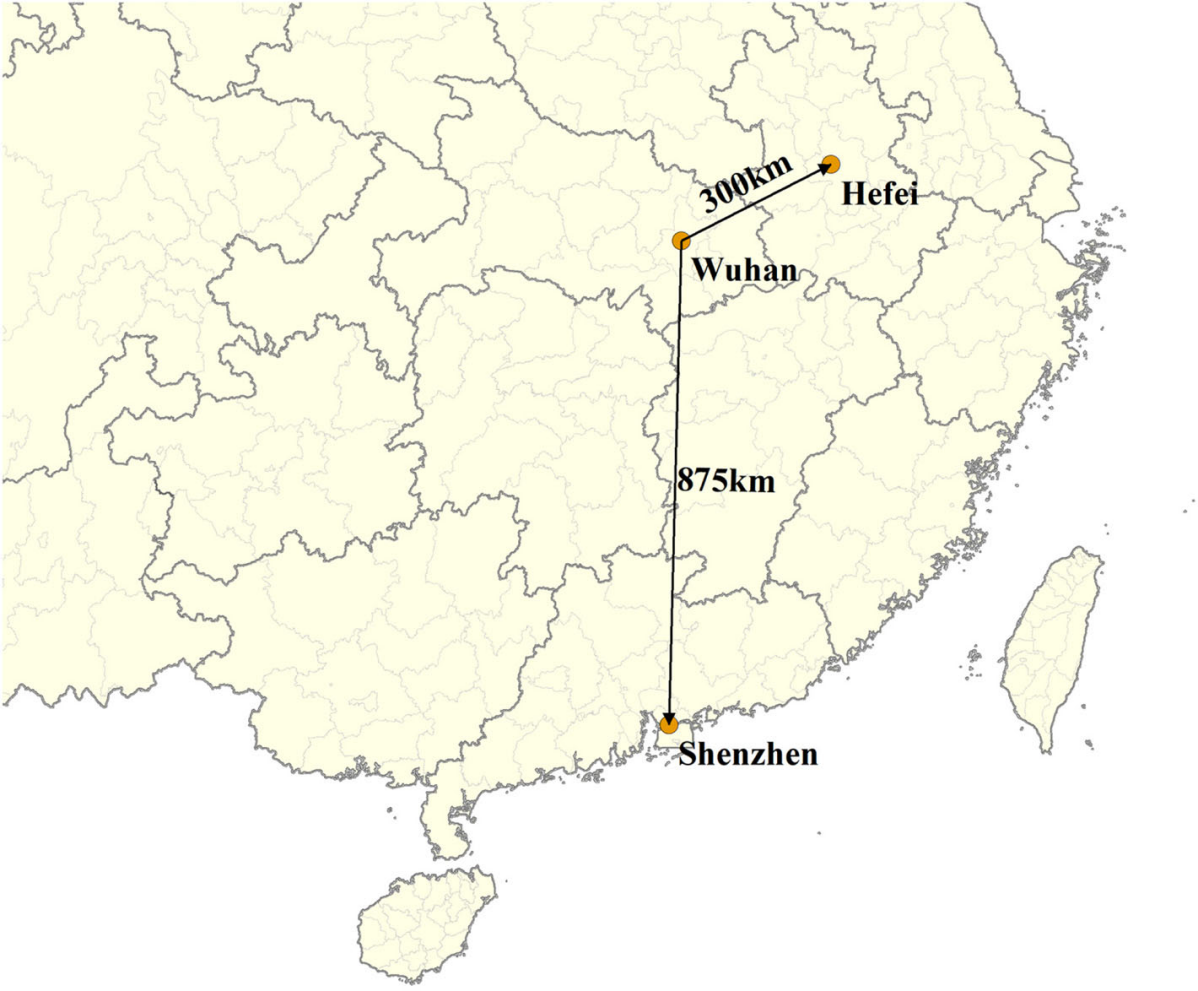
The locations of Shenzhen and Hefei in China

Shenzhen, adjacent to Hong Kong, is in the southern part of Guangdong Province in China, with a straight-line distance of 875 km to Wuhan. The latitude of this subtropical city is 22° 32′ N. According to China Meteorological Administration, the average maximum and minimum temperatures in January are 20 °C and 13 °C, respectively. The weather in January is commonly cloudy and calm. The other city, Hefei, the capital of Anhui Province, locates in the middle of China. The distance from Hefei to Wuhan is 300 km. The latitude of Hefei is 31° 86′ N, and the average maximum and minimum temperatures in January are 8 °C and 0 °C, respectively. The weather in January is commonly rainy and snowy, with higher relative humidity (Fig. 1). All the real-time temperature data for each individual at the specific date and city during the incubation period were derived from the data platform of China Meteorological Administration, which can be found at https://weather.cma.cn/.

### Data processing and methods

**Fig. 2:**
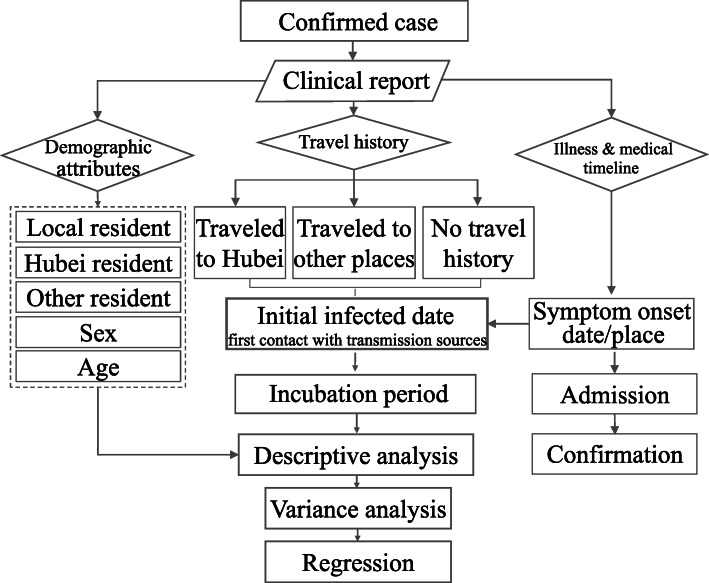
Flow chart

This analysis was based on clinical reports of confirmed patients in Shenzhen and Hefei. Basic demographic information, travel history, the date and location of symptom onset, admission date, and confirmed infection date were first collected for each patient. Then, all cases were grouped into three types according to the residence, namely, the local residents, Hubei residents, and the residents from other places. Second, according to the retrospective travel history, all cases were divided into three groups, namely, people who traveled to Hubei, people who traveled to other places, and those who did not make the travel. The grouping method would make it possible to compute the number of imported cases and cases of local community transmission in each city (Fig. 2).

Based on the travel history and residence location, we attempt to identify the incubation period for some cases. The cases of Hubei residents were first excluded because the clinical reports generally did not have clear dates of contact with transmission sources. We mainly looked at two other groups. The first group is the local residents who had clear contact records with infected persons in local communities. The second group included non-Hubei residents who had clear travel histories to Hubei or other places and became infected. For the individuals who visited and stayed in Hubei or other places for more than one day, it is challenging to define the exact date on which direct contact was made with an infected source. After careful consideration, we eventually selected the arrival date as the initial date of infection, considering the highly contagious nature of this disease [16] and lack of prevention measures taken by these individuals.

This is a limitation of the publicly-accessible data because the local public health authorities did not release more information that could help better identify contact and infected data. However, the choosing of the arrival date as the initial date of infection could be the most reasonable option as it is the earliest date that the individual could be affected, though this may potentially overestimate the length of the incubation period. After obtaining the incubation period for each patient, statistical methods including descriptive analysis, variance analysis (ANOVA), and linear regression, were employed to investigate how the incubation distribution varied between Shenzhen and Hefei and how it is related to temperature. The variance analysis and the linear regression model were implemented in the software IBM SPSS Statistics 26.0. The variance analysis was conducted in examining whether there existed a significant difference in the incubation period between Shenzhen and Hefei. All three models were estimated by the ordinary least square (OLS) method, with significant *F* statistics at the 99% level. The incubation period was treated as the dependent variable, while sex and age were treated as independent variables for model 1, city disparities were added as an independent variable for model 2, and temperature and temp-squared were added for model 3. The three models separately investigated the impact of socio-economic status, city disparities, and temperature on the incubation period. Model 1 aimed to examine the effects of demographic variables on the incubation period. Due to data availability, only age and sex could be examined in model 1. Considering the possible curvilinear relationship between age and incubation period, the quadratic term for age (age-squared) was incorporated as one explanatory variable as well. Based on model 1, a 0-1 dummy variable, city, referring to the location where the patient was reported as infected, was used as a proxy variable in model 2 to geographically test variance in the incubation period between Shenzhen and Hefei. To clarify geographical and environmental disparity effects, referring to Tan et al. [11], the variable temperature (daily average maximum environmental temperature in the place where the individual patient case first developed related symptoms) and its quadratic item were incorporated in model 3.

## Empirical results

### Demographic characteristics of infected cases

By 12 February 2020, 386 and 157 cases were reported in Shenzhen and Hefei, respectively (Table 2). Considering the permanent populations in Shenzhen and Hefei, the incidence rates were 0.035*‰* and 0.020*‰,* respectively. The number of infected cases varied slightly by sex. Shenzhen had slightly more female patients, while the number of male patients in Hefei was higher than that of female patients.

**Table 2:** Demographic information on infected cases by February 12, 2020

Variable	Shenzhen		Hefei	
	*N*	*%*	*N*	*%*
No. of cases	386	100	157	100
*Incidence ratio*	0.035‰		0.020‰	
*Age*				
≤ 10	24	6.22	2	1.27
11 ~ 20	16	4.15	4	2.55
21 ~ 30	33	8.55	29	18.47
31 ~ 40	87	22.54	30	19.11
41 ~ 50	61	15.80	41	26.11
51 ~ 60	71	18.39	29	18.47
61 ~ 70	80	20.73	12	7.64
≥ 71	16	4.15	10	6.37
*Sex*				
Male	184	47.67	84	53.50
Female	202	52.33	73	46.50
*Residence*				
Local	234	60.62	116	73.89
Hubei	136	35.23	28	17.83
Other places	15	3.89	11	7.01
*Travel history*				
Hubei	287	74.35	42	26.75
Other places	30	7.77	16	10.19
No travel history	68	17.62	97	61.78

**Fig. 3:**
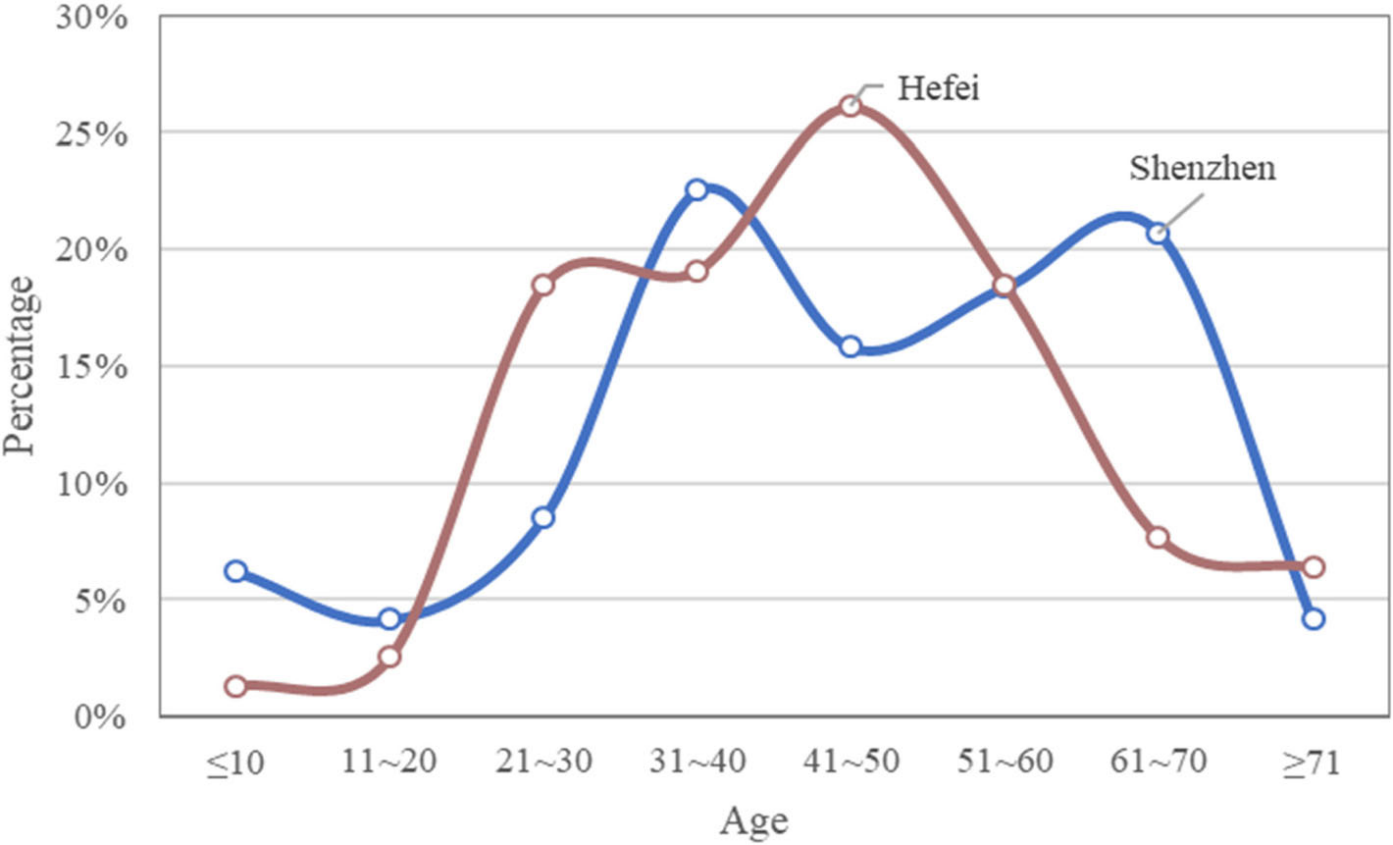
Association between age and the number of infected cases

In contrast to the slight variation by sex, the number of patients varies with age (Fig. 2). In general, an inverse U-shaped relationship possibly exists between age and the number of infected cases reported in Shenzhen and Hefei. Namely, compared to children and the elderly population, more infected cases are adults between the ages of 30 and 50 years. This feature is evident in Hefei, where the largest proportion of cases is in the age interval from 41 to 50 years. In Shenzhen, there were two peaks in the age distribution. The first is the age group from 31 to 40 years, and the second is the age group from 61 to 70 years (Fig. 3).

These two cities contain their own demographic features that potentially lead to the difference. As the largest immigrant city in China, Shenzhen has a large proportion of young residents (approximately 30 years old) who traveled to their hometown in Hubei Province for family reunions during the Spring Festival holiday and were finally reported as infected when they came back to Shenzhen. At the same time, a large number of potentially infected elderly patients living in Hubei Province came to Shenzhen to celebrate the Spring Festival holiday with their children. According to the data, 43.8% of all 137 infected cases who resided in Hubei and traveled to Shenzhen were over the age of 60 years. Hence, the percentage of infected patients who live in Hubei Province but were reported as infected in the city of Shenzhen is high. In contrast, the largest number of infected cases in Hefei was the local residents who did not have travel histories. This indicates that most cases were likely to be infected by human-to-human transmission within local communities in Hefei.

### Symptom onset location

**Fig. 4:**
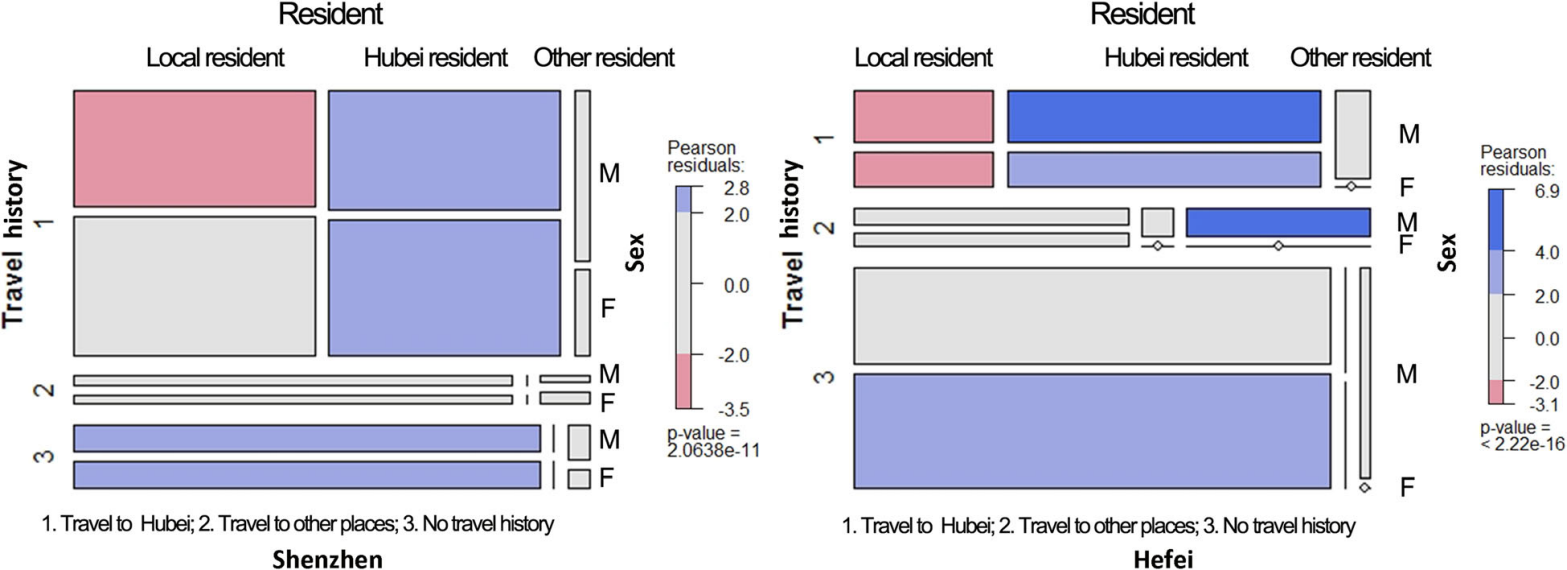
Demographic structure of infected cases in Shenzhen and Hefei

**Fig. 5:**
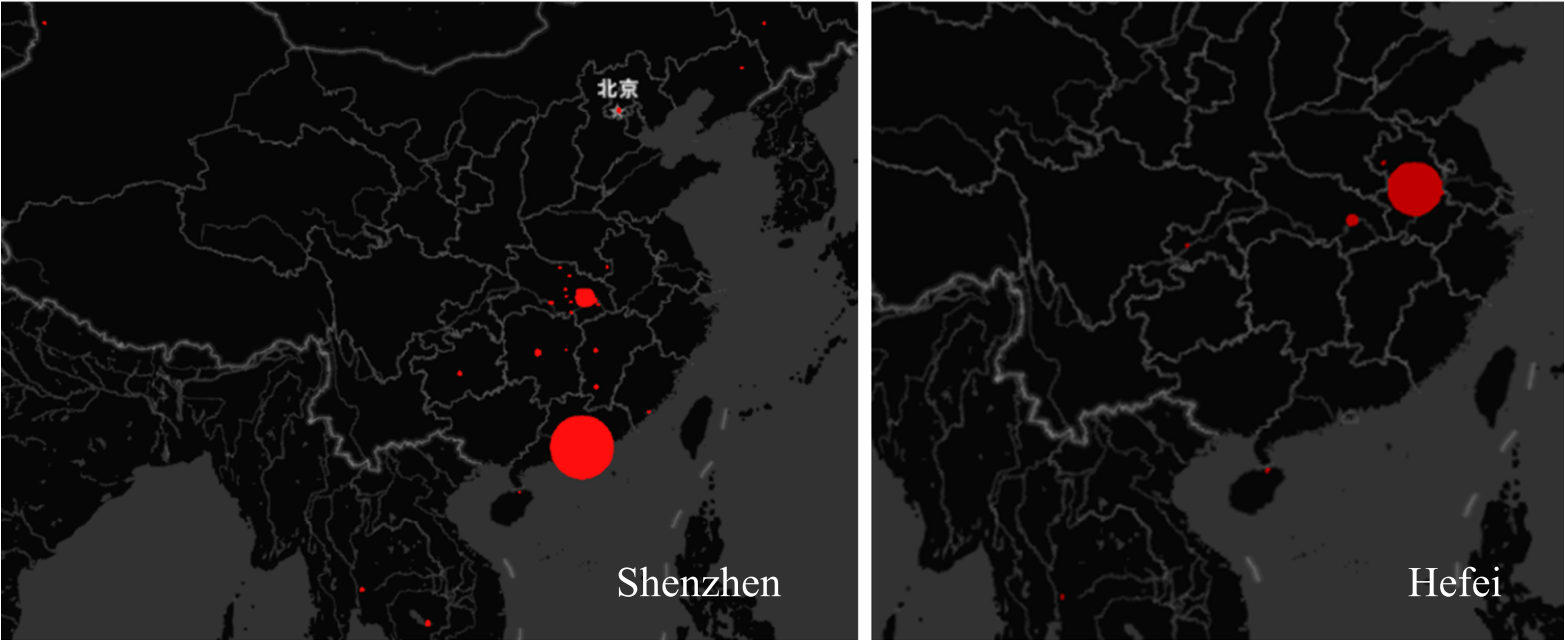
Geographic distribution of symptom onset locations

Eighty percent of patients confirmed in Shenzhen or Hefei first exhibited symptoms in Shenzhen and Hefei. However, some patients were reported to have initial symptoms in Hubei and other places. Forty-eight patients reported in Shenzhen and 14 patients in Hefei had relevant symptoms first in Hubei (Table 3). Among them, 37 patients lived in Hubei, 31 patients finally traveled to Shenzhen, and 6 patients traveled to Hefei (Fig. 4). On the other hand, 43 patients exhibited related symptoms in other places (beyond Shenzhen, Hefei, and cities in Hubei Province). These places even include some cities outside China, i.e., Phuket Island in Thailand, Yangon, in Myanmar, London, and Rome (Fig. 5). These infected cases have become traveling transmission sources, substantially enlarging the infected areas worldwide.

**Table 3:** Distribution of symptom onset locations

Symptom onset location	Shenzhen		Hefei		Total	
	*N*	*%*	*N*	*%*	*N*	*%*
Local city	305	79.01	132	84.07	437	80.62
Hubei	48	12.44	14	8.92	62	11.44
#Hubei resident	31		6		37	
Other places	33	8.55	11	7.00	43	7.93

### Variance analysis of the incubation period

Although all patient cases had detailed records of the symptom onset date and location, the initial infection date could be identified for only 176 cases reported in Shenzhen and 41 cases in Hefei (Table 4). A total of 64.7% of the 217 cases were the local residents in Shenzhen and Hefei who traveled to Hubei. Among all patients, the average incubation period was 8.58 days, and the median value was 8 days.

**Table 4:** ANOVA on the incubation period between infected cases in Shenzhen and Hefei

Variable	Shenzhen	Hefei	Total
No. of infected cases with a clear incubation period			
No. of infected cases	176	41	217
Proportion of all infected cases	45.59%	26.11%	39.96%
#Traveled Hubei	139	12	141
#Traveled to other places	12	4	16
#No travel history	25	25	50
Normal testing			
Median	9	4	8
Mean	9.27	5.61	8.58
Std. dev.	4.59	3.62	4.65
95% *Conf. interval*	[8, 10]	[4, 6]	[7, 9]
Skewness	0.324	0.938	0.424
Std. error	0.183	0.369	0.165
Kurtosis	−0.543	0.161	−0.534
Std. error	0.364	0.724	0.329
*F* testing			
*F*	22.671		
Prob > *F*	0.000		

**Fig. 6:**
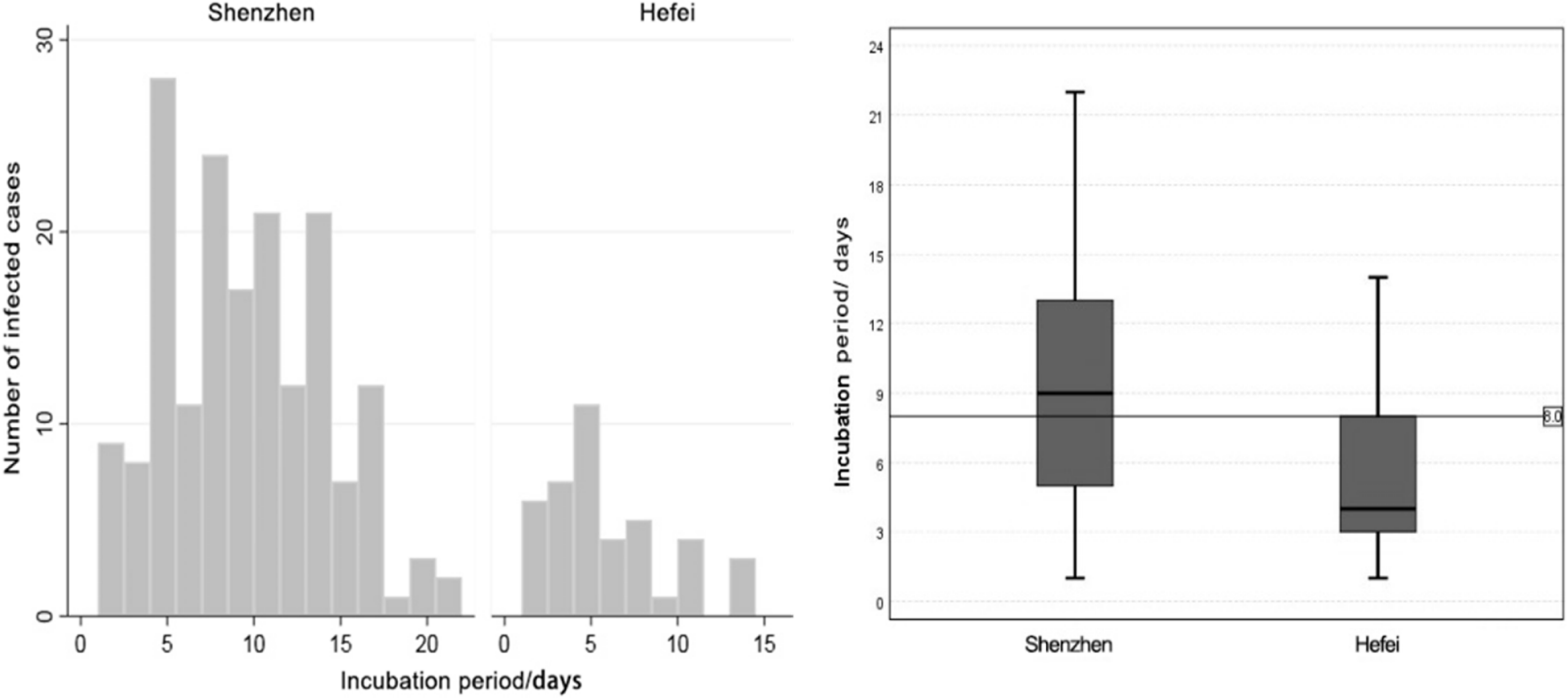
Incubation period distributions for infected cases reported in Hefei and Shenzhen

It is notable that the incubation period varied between Shenzhen and Hefei. Specifically, the median was 9 days for cases reported in Shenzhen and 4 days in Hefei. The interquartile range was from 5 days and 13 days in Shenzhen and from 3 days to 8 days in Hefei. The kurtosis Z score of the incubation time is calculated separately as − 1.483, − 1.507, 0.447 for the whole, Shenzhen, and Hefei, which are all within [− 1.96, 1.96]. After the normal testing, a one-way analysis of variance indicated that differences in the incubation periods across two cities are statistically significant at the 99% level (Fig. 6). The Kruskal-Wallis test also presents a significant result. This suggests there exist geographical disparities in the incubation period for patients of the cities.

### Linear regression on the incubation period

Since the variable of incubation period basically fits the normal distribution (Table 4), this variable was directly used as the dependent variable in the following models (Table 5). The *R*-squared value of model 1 was as low as 0.075, indicating that the power of the demographic variables for explaining the variance in the incubation period is not adequate. However, the *R*-squared value increased steadily after incorporating location disparity factors in model 2 (0.135) and geo-environmental factors in model 3 (0.208). For limited pathogenesis-related variables, the *R*-squared values for both models, to some extent, were acceptable.

**Table 5:** Regression results for the geographical disparities in the incubation period

Variable	Model 1		Model 2		Model 3	
	*Coef.*	*p*	*Coef.*	*p*	*Coef.*	*p*
Constant	13.331	0.000	13.325	0.000	9.662	0.000
Sex	0.191	0.755	−0.032	0.957	−0.047	0.933
Age	−0.297	0.000	−0.258	0.000	−0.250	0.000
Age squared	0.003	0.000	0.003	0.000	0.003	0.000
City^a^			−3.313	0.000		
Temp^b^					0.717	0.001
Temp-squared^b^					−0.030	0.000
No. of obs.	217		217		217	
Prob > F	0.000		0.000		0.000	
*R* squared	0.075		0.151		0.226	
Adj. *R* squared	0.062		0.135		0.208	

^a^The baseline is Shenzhen; this variable was deleted from model 3 because of collinearity

^b^Average maximum temperature in the place where the patient first developed related symptoms

First, the factor of sex does not have a statistically significant linkage with the variance in the incubation period in any models. This outcome is in line with the finding that no evident differences exist based on sex concerning the number of infected cases.

Second, the coefficient for the effect of age and its quadratic term (age-squared) on the incubation period are − 0.258 and 0.003, respectively. Both estimations were statistically significant at the level of 99%. Although the positive elasticity in the quadratic term of age is very small, it, together with the negative elasticity in age, statistically confirms that a U-shaped relationship exists between age and incubation period [17].

Third, the increasing *R*-squared values from model 1 to model 3 support the hypotheses regarding the geographical disparities in the incubation period. By designing model 2, we examined how geographical/locational disparities can affect the incubation period, as the city itself could lead to a difference in the incubation period. The shorter incubation period for patient cases in Hefei was statistically verified. Temperature, as a key geo-environmental element, was designed into model 3 in exploring whether the temperature of the cities is associated with the incubation period. Thus, we ran a stepwise regression involving the variable of temperature for model 3, where the variable of the city was excluded from the model but with a significant increase of R square. According to the results of model 3, the coefficient for the variable of temperature is positive (0.717), while the coefficient for the quadratic term (temp-squared) is negative (− 0.030). Both elasticities are statistically significant at the level of 99%. The increase of *R* square from model 2 to model 3 proved that the temperature of cities (geo-environmental disparities) better explained the association with the incubation period than location disparities.

## Discussion

**Fig. 7:**
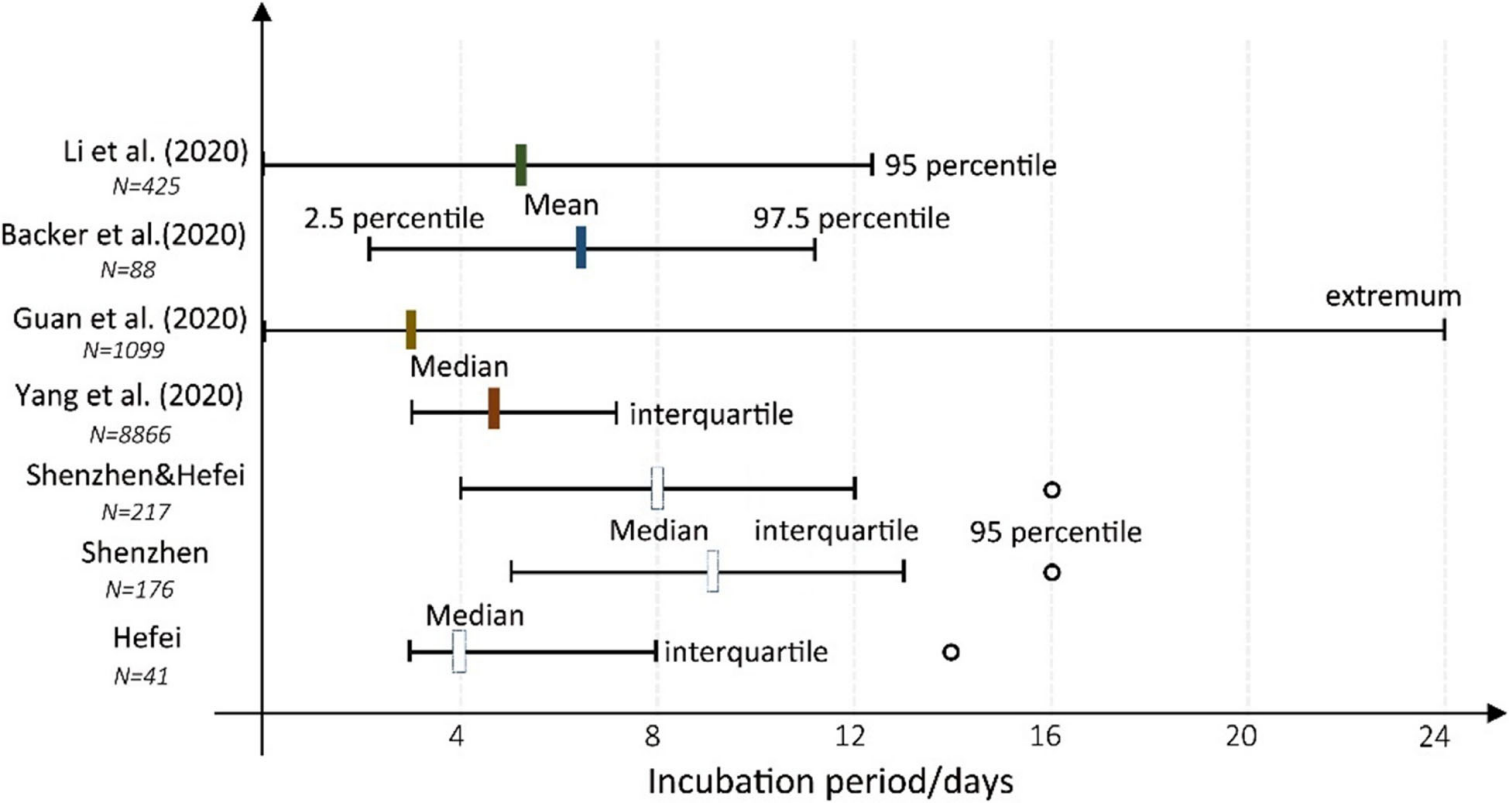
Incubation periods of COVID-19 from different studies

As one of the critical parameters for understanding the COVID-19 epidemics, incubation periods are being continually updated as an increasing number of clinical case records have been obtained. As shown in Fig. 7, the outcome varies across the cases used in each study. Li et al. first declared that the mean incubation period was 5.2 days among the 425 infected patients identified early in the epidemic, and the value at the 95th percentile of the distribution was 12.5 days [5]. Using the 88 early cases with travel histories to Wuhan, Backer et al. found that the mean incubation period was 6.4 days, ranging from 2.1 (2.5th percentile) to 11.1 days (97.5th percentile) [7]. Nevertheless, the findings obtained from some large-size case studies were slightly conservative in that the incubation period had a lower median and broader range. For instance, based on 1099 patient cases, Guan et al. concluded that the incubation period ranged from 0 to 24 days, and the median was 3 days [6]. In this study, the median (8 days) for 176 valid patient cases was larger than in other studies. Only for the 41 cases reported in the city of Hefei, the median value was 4 days, less than the incubation periods reported in other studies (Fig. 7). Hence, the variance probably did not result from the number of cases in each study. The problem partly lies in the feature of patient cases. The early patient cases, which resided in Wuhan which was mainly studied by current studies, probably were infected with the early-generations virus that has more substantial virulence. Along with human-to-human transmissions to local communities in Shenzhen, the virulence of the virus on the third-or-fourth generations of cases may decrease and lead to a shorter incubation period.

In addition, the methods of identifying the incubation period may also lead to some bias. According to Backer et al., the incubation period was determined as the duration from the date when the case left Wuhan to the date of illness onset [7]. As the authors admitted, this determination may be slightly conservative. In contrast, the incubation period in this study is determined from the date the patient arrived in Hubei to the illness onset day. Also, publicly reported cases may overrepresent severe cases, the incubation period for which may differ from that of mild cases. So the incubation period could be slightly overestimated. The exact date of infection is between the initial contact date and the last contact date.

The incubation period is averagely longer than the evidence from (sub)tropical countries, 6.93 days for India [18] and 5-6 days for Indonesia [19]. This might be due to the limitation we chose arrival date as the beginning of the incubation period, which could overrepresent those who got infected at the later period of their staying in Wuhan. Though some research through macro-level analysis indicated there is no evidence supporting that case counts of COVID-19 could decline when the weather becomes warmer [20]. The choosing of the initial date arriving Wuhan might potentially overestimate the length of the incubation period, but it would not neglect the fact that the individual might get infected at any time during his/her stay in Wuhan.

It is still under discussion, especially when engaging the big data method. The big data released by local public health authorities sometimes lack detailed information, and methodology based on big data technology could also contribute to the understanding of epidemiological elements of COVID-19. Another reason why we insisted on adopting this definition is that the travels on the part of patients who had been to Hubei in this study generally occurred after January 10, 2020, unlike the cases in Backer et al., who traveled from Wuhan during the initial stage of this epidemic [7]. In light of the extremely rapid transmission of this virus along with the pre-Spring-Festival travel rush and the fact that the infected patients did not usually take any preventive measures before January 22, 2020, it is assumed the risk of being infected after arriving at Hubei was high. Therefore, we defined the incubation period as described above in this study, given the fact that there are not any more commonly accepted methods for big data usage. With more updated and detailed data, the following research could just investigate the incubation period for cases that have clear epidemical contact date no matter the cases were imported ones or were infected by transmission within local communities.

Compared to calculating the specific values of the incubation period, the focus of this research was to look at the geographical disparities in the incubation period distribution. After employing several statistical methods, this study sufficiently demonstrated that the incubation periods of COVID-19 vary across the patients reported in two cities, Hefei and Shenzhen, China. The infected cases in Shenzhen averagely had more extended incubation periods. From the perspective of medical geography, we attempt to attribute this geographical variability to the variances in the maximum temperature of the outdoor daytime before the date of illness onset for each individual, and the explanatory power of which reached 15% in this study. We further infer that under the warmer condition in the winter of Shenzhen, the multiplication and transfer of this novel coronavirus within the human body may decrease. The decrease of viral virulence probably decelerates the process of cell lesions and immune injury, and then postpones the incubation duration from being infected to the clinical symptom onset. On the contrary, a comparatively colder environment in Hefei, could result in physiological responses and weaken the function of the human immune system [21, 22], which in return may raise the risk of being infected and leads to a short incubation period. Worthy to mention, the U-shaped relationship exists between age and incubation period suggests that adults are probably more vulnerable to this coronavirus after infection, resulting in a shorter incubation period in adults. Considering that the largest proportion of infected cases are adults from 30 years to 50 years, persons in this age interval should be more cautious about the risk of being infected.

The association between viral virulence and environmental temperature for COVID-19 probably is in line with Chan et al. and Casanova et al. [12, 13], which revealed the stability of the SARS virus at different temperatures and relative humidity by laboratory experiments and also reached a consistency with Yang et al. and Qin et al. [23, 24] using COVID-19 empirical cases. To further verifying this process, subsequent studies or experiments could consider incorporating more environmental indicators, i.e., relative humidity and ultraviolet intensity, subject to data availability.

## Conclusion

Understanding the incubation period of COVID-19 is crucially important in the face of the ongoing epidemic [25]. Based on the clinical reports of infected cases in Shenzhen and Hefei, this contradistinctive study determined the incubation period for each patient and disclosed the geographical disparities in the incubation period distribution of this novel coronavirus. In particular, the median incubation period for infected cases in Shenzhen was 9 days, with an interquartile range from 5 to 13; while in Hefei, the median incubation period was 4 days, with an interquartile range from 3 to 8. Through individual-based micro-level analysis, we found the incubation period has an inverse U-shaped numeric relationship with the maximum temperature of the outdoor environment. In this study, when the meteorological temperature was maintained at approximately 10 ~ 15 °C, the incubation period became the longest.

Profiling the incubation distribution in geographical scale can benefit policymakers in determining the appropriate quarantine duration for different regions and evaluating how many days should be included in the tracing of contacts for both infected cases and suspected cases [8, 26]. Acknowledging the association between environmental temperature and the incubation period facilitates the forecasting of the transmission and evolution cycle. It is also encouraged that the local public health authorities at the different administrative levels can share brief clinical reports information for epidemics like COVID-19, especially at the early stage of epidemic spread. The utilization of methodologies based on big data and open sources can energize the research on the epidemiological elements and in turn support governments’ policymaking process.

## Data Availability

The data is publicly accessible from local public health authorities, which also have been uploaded to the submitting system.
